# Management of Crohn's disease

**DOI:** 10.1136/archdischild-2014-307217

**Published:** 2015-11-09

**Authors:** Jochen Kammermeier, Mary-Anne Morris, Vikki Garrick, Mark Furman, Astor Rodrigues, Richard K Russell, BSPGHAN IBD Working Group

**Affiliations:** 1 Institute of Child Health, Great Ormond Street Hospital, London, UK; 2 Department of Paediatrics, Norfolk and Norwich University Hospital, Norwich, UK; 3 Department of Paediatric Gastroenterology, Royal Hospital for Children, Glasgow, UK; 4 Centre for Paediatric Gastroenterology, Royal Free Hospital, London, UK; 5 Department of Paediatric Gastroenterology, John Radcliffe Hospital, Oxford, UK

**Keywords:** Gastroenterology, Therapeutics

## Abstract

Crohn's disease (CD) is rapidly increasing in children so an up to date knowledge of diagnosis, investigation and management is essential. Exclusive enteral nutrition is the first line treatment for active disease. The vast majority of children will need immunosuppressant treatment and around 20% will need treatment with biologics. Recent guidelines have helped make best use of available therapies.

## Introduction

Crohn’s disease (CD) is a chronic inflammatory disorder, which can affect any part of the intestinal tract as well as extraintestinal tissue. Factors that contribute towards the pathogenesis of the disease are the host's genetic profile, immune system and environmental factors such as the gut microbiota.^1^

CD occurs in all ages with recently reported paediatric figures from Scotland and South England suggesting an incidence of 4.75 and 5.85/100 000 people, respectively.^2^ ^3^ UK prevalence data are limited, but suggest a figure of 32 per 100 000 people.^4^ Due to yet undefined environmental factors, the incidence and prevalence of CD is rising, in both adult and paediatric studies.^5^ ^6^

In this review, we aimed to update important new developments in the management of CD for paediatricians in the UK highlighting the key publications and guidelines published recently.^7^ We also recognise that the BSPGHAN inflammatory bowel disease (IBD) guidelines published previously, now contain many recommendations that needed updated guidance, but without generation of a specifically new guideline.^8^ ^9^

### Diagnosis

**Figure 1:**
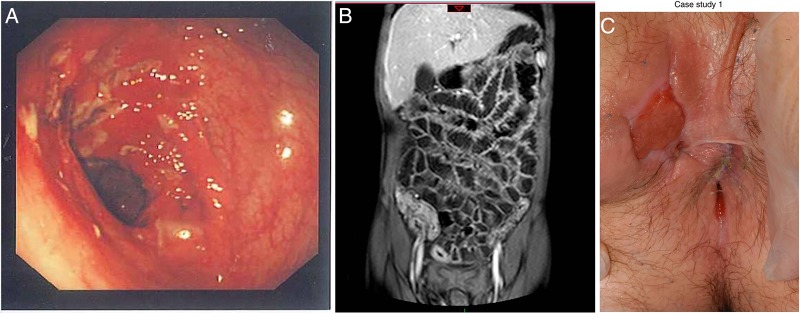
(A) Left: endoscopic view of the transverse colon in Crohn’s disease (CD) revealing a cluster of deep ulcers. (B) Centre: magnetic resonance enterography showing extensive terminal ileal disease. (C) Right: perianal disease (inflamed skin tag and external orifice of a fistula at 10:00) in CD patient (reproduced with permission).

Key clinical symptoms of CD comprise diarrhoea, abdominal pain, growth failure and rectal bleeding with the first three being the most common in patients first presenting with CD.^10^ CD often has an insidious onset, which may contribute to considerable diagnostic delay. CD can affect the entire intestinal tract, and transmural inflammation can lead to stricture formation and fistulisation between the gut and other abdominal organs as well as the skin.^11^ Perianal inspection is imperative as significant perianal involvement (eg, inflamed fissures or skin tags, abscesses and fistulae) are seen at presentation in at least 15% of children with CD^12^ (see figure 1). Extraintestinal manifestations in IBD can affect the skin, eyes, musculoskeletal and hepatobiliary systems.^13^

**Figure 2:**
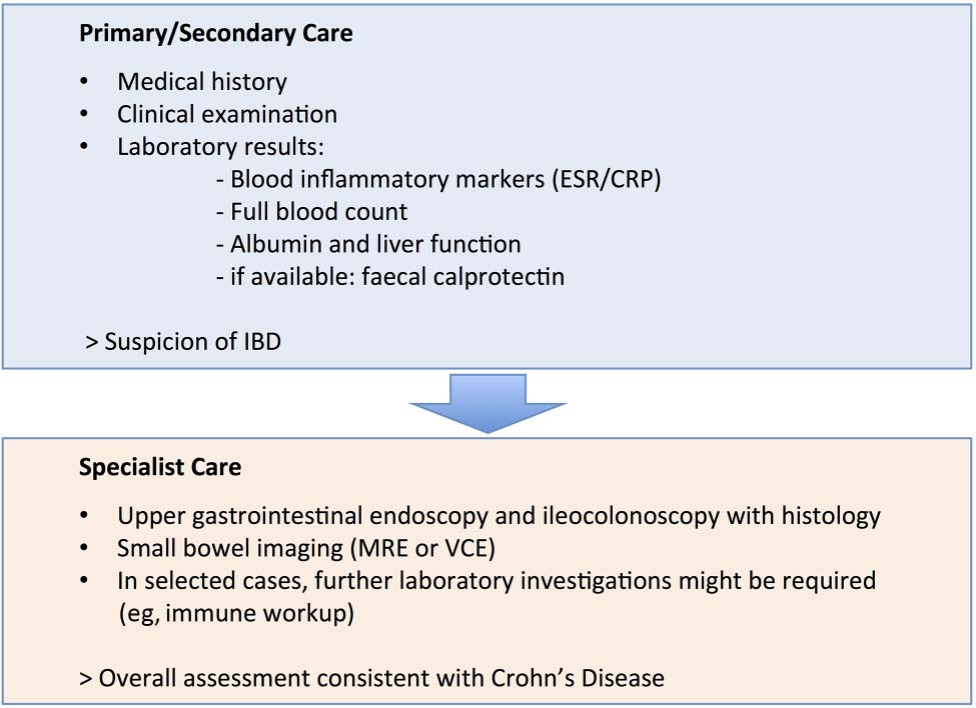
Diagnostic pathways in Crohn’s disease (CD). Patients displaying features, consistent with CD should be fast tracked for specialist review. IBD, inflammatory bowel disease; MRE, magnetic resonance enterography; VCE, video capsule endoscopy.

Once IBD is suspected, patients should be fast tracked to specialist services (for relevant blood and stool tests see Fell *et al*13a and figure 2). The revised Porto criteria provide detailed guidance on the diagnostic evaluation of paediatric patients with IBD^10^ emphasising the necessity to perform upper gastrointestinal endoscopy and ileocolonoscopy with histology as well as small bowel imaging (see figure 1).^14^

Once CD has been established, ongoing disease activity is monitored by clinical review and assessment. The originally reported Paediatric Crohn’s Disease Activity Index (PCDAI)^15^ is now mostly superseded by the weighted PCDAI which takes clinical symptoms, laboratory markers, anthropometric data and clinical examination into account (table 1).^16^ This is an assessment, which can be used by all paediatricians to objectively assess disease activity.

**Table 1:** The weighted Paediatric Crohn's Disease Activity Index^16^

			Score
*History (recall, 1 week)*			
Abdominal pain			
**0**=none	**10**=mild: brief, does not interfere with activities	**20**=moderate/severe: daily, longer lasting, affects activities, nocturnal	______
Patient functioning, general well-being			
**0**=no limitation of activities, well	**10**=occasional difficulty in maintaining age appropriate activities, below par	**20**=frequent limitation of activity, very poor	______
Stools (per day)			
**0**=0–1 liquid stools, no blood	**7.5**=up to 2 semiformed with small blood, or 2–5 liquid	**15**=gross bleeding, or ≥6 liquid or nocturnal diarrhoea	______
*Laboratory*			
Erythrocyte sedimentation rate			
**0**≤20 mm/h	**7.5**=20–50 mm/h	**15**≥50 mm/h	______
Albumin			
**0**≥3.5 g/dL	**10**=3.1–3.4 g/dL	**20**≤3.0 g/dL	______
*Examination*			
Weight			
**0**=weight gain or voluntary weight stable/loss	**5**=involuntary weight stable, weight loss 1%–9%	**10**=weight loss ≥10%	______
Perianal disease			
**0**=none, asymptomatic tags	**7.5**=1–2 indolent fistula, scant drainage, no tenderness	**15**=active fistula, drainage, tenderness or abscess	______
Extraintestinal manifestations			
fever ≥38.5°C for 3 days over last week, definite arthritis, uveitis, erythema nodosum and pyoderma gangrenosum			
**0**=none		**10**=one or more	______
Total score (0–125)			

Remission <12.5; mild 12.5–40; moderate 40–57.5; severe disease activity >57.5.

## Treatment strategy

Treatment aims are to induce and maintain clinical remission, optimise nutrition, define bone status, optimise growth and pubertal progress and minimise drug adverse effects. Treatment beyond simple symptom resolution is now commonly employed with intestinal/mucosal healing the target of contemporary CD care.^17^

The strategy of early introduction of immunomodulatory and biological therapies to induce deep remission (long-term intestinal healing without relapse) particularly in high-risk patients (severe endoscopic or perianal disease, poor response to induction therapy, extensive, stricturing or penetrating disease, marked growth retardation and/or severe osteoporosis) might change the natural history of the disease and is being increasingly used in paediatric IBD.

## Induction of remission

### Exclusive enteral nutrition

Exclusive enteral nutrition (EEN) for 6–8 weeks, using a whole protein formula is the first-line therapy to induce remission in children with active CD.^7^ Clinical response rates are approximately 80% and similar to corticosteroids.^18^ ^19^ However, in contrast to corticosteroids, EEN offers significant nutritional advantages and provides superior mucosal healing in comparison to steroids.^20^ ^21^ Clinical improvement usually occurs within days and alternative therapy should be considered in the absence of response within 2–4 weeks. No differences between polymeric (whole protein) and elemental (amino acid) formulae have been shown.^22^ ^23^ Polymeric feeds are the preferred option as they are better tolerated, more cost-effective and require less nasogastric feeding.^24^

Success with EEN is maximised by robust and frequent patient and family support from the multidisciplinary team (MDT), particularly in the early stages of treatment. Clinical review halfway through therapy further facilitates completion of the full course.^25^ Limited data suggest that ongoing maintenance enteral nutrition can be a useful strategy in some children after the course of exclusive enteral nutrition is complete.^26^

### Corticosteroids

Corticosteroids can be used to induce remission in CD, if EEN is not possible or the patient does not respond to therapy. Prednisolone is given orally (1 mg/kg, maximum: 40 mg/day, tapering over 8–10 weeks) for moderate/severe active luminal CD. For mild/moderate ileocaecal disease, budesonide is an alternative option with fewer steroid-related side effects (maximum: 12 mg/day, tapering over 2–4 weeks). Intravenous steroids (preferred intravenous steroid: methylprednisolone at 1–1.5 mg/kg, maximum: 60 mg/day; or alternatively: hydrocortisone 2–4 mg/kg/dose, maximum 100 mg/dose four times a day) may be initially needed for severe disease.

Similar to EEN, clinical remission rates of up to 80% have been reported, but with considerably lower mucosal healing rates.^27^ ^28^ Adverse effects are proportional to dose and duration, and include adrenal suppression, growth failure, cosmetic and behavioural effects.

### Anti-tumour necrosis factor α antibodies

Anti-tumour necrosis factor (TNF) therapy is also recognised as induction therapy for selected patients with moderate to severe disease. These therapies will be discussed in detail in the Maintenance of remission section.

### Antibiotics

Adult studies demonstrating reduced fistula drainage support the use of antibiotics (metronidazole and/or ciprofloxacin) in perianal fistulising CD disease.^29^ Limited paediatric data on combination treatment with azithromycin and metronidazole in luminal disease have been encouraging, but await validation in larger better designed clinical trials.^30^

## Maintenance of remission

### Thiopurines

Results from paediatric and adult studies support the use of thiopurines (6-mercaptopurine or azathioprine) to maintain disease remission.^27^ ^31^ At 18 months, cumulative steroid doses and relapse rates were significantly lower in children on 6-mercaptopurine compared with placebo (9% vs 47%). In addition, a reduced need for surgery in CD was reported in patients on azathioprine.^32^ These benefits need to be balanced against the side effects and long-term risks of immunosuppressant therapy.^33^ For detailed management of azathioprine see Fell *et al*.13a

### Methotrexate

Paediatric retrospective cohort studies report 50%–80% effectiveness of methotrexate (MTX) in children who failed to respond or were intolerant to thiopurine therapy in maintaining remission.^34^ ^35^

MTX (15 mg/m^2^ one time per week, maximum: 25 mg subcutaneously) in conjunction with folate supplementation is an alternative option as primary maintenance therapy or following thiopurine failure or intolerance.^7^

MTX is particularly suitable as first-line treatment in patients who have coexistent inflammatory arthritis.^36^ Data on oral bioavailability are variable, but the latest evidence suggests that initiating treatment subcutaneously followed by administering the drug orally can be an alternative to long-term subcutaneous administration.^37^ Adverse events include flu-like symptoms, transaminitis and infrequently myelosuppression, which may require dosage adjustment or drug withdrawal. Significant hepatocellular liver disease is rare. Nausea and vomiting have been reported in 11%–24% of patients, and can be successfully controlled with antiemetic medication in many but not all patients.^38^ Contraception is essential. Teaching patients and families to deliver the drug in the home setting is achievable for the majority, allowing flexibility in drug delivery, increasing drug acceptability while reducing overall cost.^39^

### Anti-TNFα antibodies

Prior to commencing anti-TNF therapy (chimeric monoclonal antibody: infliximab; or humanised monoclonal antibody: adalimumab), patients have to be screened for tuberculosis and hepatitis B to prevent reactivation. Varicella immunity should also be established and in seronegative cases, and if feasible, varicella zoster immunisation should be considered before treatment.

Anti-TNF treatment is recommended for inducing and maintaining remission in children with steroid refractory disease or CD affecting the gut lumen (luminal disease) despite optimised immunomodulation. Anti-TNF therapy is also used by some clinicians as primary induction therapy in active perianal disease and in patients at risk of poor outcomes, but both indications are currently outside of the drugs licence.^7^

In luminal disease, infliximab response rates of up to 90% and remission rates at 1 year of 55%–60% have been reported.^40^ ^41^ Infliximab improved outcomes of perianal CD with response rates of 75% and remission rates of 50% at 1 year.^42^ Adalimumab achieved 1-year remission rates of 45% in anti-TNFα antibody naïve children and 20% in infliximab non-responders.^43^

Infliximab is first administered intravenously in doses of 5 mg/kg at weeks zero, two and six followed by 8-weekly infusions. Adalimumab is administered subcutaneously on alternate weeks (most commonly 40 mg) after initial loading dose(s) of 80 or 160 mg.

The proportion of primary anti-TNF non-responders (failure to respond after induction course of 6 weeks) in paediatric CD is low (10%–25%). However, more commonly antibody formation against the drug over time can result in secondary loss of response.^44^ The formation of antibodies and the resulting loss of response are reduced by the use of co-immunosuppression with either thiopurines or MTX.^33^ ^45^ As such the strategy of combination therapy is commonly employed in the UK, but this needs to be continuously balanced against the increased side effect profile of this strategy. Recently, infliximab drug and antibody levels have become available to support decision making in partial or complete lack/loss of treatment response.^46^ ^47^ If antibody and drug trough levels are inadequate, dose escalation and/or interval reduction can regain response.^7^

Adverse effects following anti-TNF therapy include acute infusion reactions (up to 15%), delayed hypersensitivity reactions (8%), skin eruptions (eczema/psoriasis, 8%) and serious infections (3.3%) including reactivation of latent tuberculosis.^48^

### Other treatments

Inducing remission with aminosalicylates may be considered rarely in mild colonic CD in a small subset of patients only. The evidence for other immunomodulators is not as extensive as for thiopurines and MTX, but thalidomide, sirolimus and tacrolimus can be useful in selected patients’ dependant on individual circumstances.^49–51^

### Surgery in CD

The risk of requiring surgery within 5 years of diagnosis in paediatric CD is around 20%.^52^ In one retrospective study, out of 69 patients who required surgery, 58% underwent right hemi-colectomy, 3% subtotal colectomy, 12% stoma formation and 14% surgery for perianal disease.^53^

Patients with late-onset paediatric, severe/extensive or stricturing/penetrating CD have increased the risk for bowel surgery. Emergency surgical interventions are indicated in circumstances such as severe haemorrhage, intestinal perforation and toxic megacolon.

Otherwise, surgery is commonly carried out in three scenarios: isolated/localised disease including perianal disease, IBD refractory to conventional medical therapy and when complications arise such as strictures and fistulae.^53^ ^54^ Resectional surgery is increasingly being carried out laparoscopically.

In cases where CD is limited to an isolated bowel segment (eg, ileocaecal CD), surgery might establish a postoperative recurrence-free interval to allow for growth and development.^55^

Bowel rest through temporary diversion ileostomy has been considered in patients with extensive colonic and/or perianal disease refractory to medical treatment. Treatment of perianal fistulising CD with anti-TNF therapy is effective and significantly reduces the need for surgery.^56^ Combined medical and surgical treatment within the MDT is regarded by many as the best treatment approach to complex perianal disease.^57^ Enteroenteric fistulae may respond to anti-TNF therapy, but are more likely to require surgical intervention. Perianal fistulae can be transsphincteric, intersphincteric, suprasphincteric, extrasphincteric or subanodermal. Fistulae management requires an individualised approach considering surgical and medical options. Surgical fistulotomy in patients with a simple, symptomatic fistula is considered appropriate. Surgery in complex fistulae bares a high risk of sphincter damage and faecal incontinence.^58^ ^59^ Insertion of loose, ie non-cutting seton sutures after incision and drainage can facilitate better long-term healing, and prevention of recurrence of abscesses in selected patients with perianal disease.^59^

Strictures particularly involving the terminal ileum/ileocaecal valve are a common indication for CD surgery.^52^ Limited resections or stricturoplasty are considered best practice to preserve gut length and prevent short bowel syndrome.

## General considerations

### IBD and cancer

The overall risk to develop malignancies such as lymphomas for children with IBD is low, but higher than the general paediatric population. In one large cohort study of paediatric IBD patients, two lymphomas were diagnosed in a 30-year period (the risk of developing lymphoma on thiopurines was found to be approximately eightfold higher when compared with the general population).^60^ Over 30 cases of hepatosplenic T-cell lymphoma, which is associated with very high mortality and mainly affects young men, have been reported in IBD patients. The majority (56%) were treated with anti-TNF–thiopurine combination therapy.^61^ Mortality overall is very rare in paediatric IBD and usually is secondary to infection or cancer.^62^

### Vaccination

Live vaccination (eg, varicella) should be considered in immune competent IBD patients prior to long-term immunosuppression. The annual inactivated influenza vaccination should be offered to all children with IBD too.^63^

### Education

Children and their parents should be encouraged to engage with national and international patients’ IBD forums such as CICRA (‘Crohn's in Childhood Research Association’) (http://www.cicra.org) and ‘Crohn's and Colitis UK’ (http://www.crohnsandcolitis.org.uk).

### Multidisciplinary team

National standards dictate that children and young people with IBD are managed, at least in part, by a Paediatric Gastroenterology team including medical, nursing and dietetic staff.^64^ ^65^ In this age group, disease control and maintaining patient engagement can be challenging and appropriate support to the patient and carers is vital.

### Transition

Transition to adult services is a particularly challenging time for the patient and family. It is best done as a staged process over months to years dependent on the physical, psychological and emotional state of the young person and should prepare them for managing their consultation independently.^66^ ^67^ Continuity and consistency are recommended to encourage engagement in transition, and the clinical nurse specialist working within the MDT often fulfils this role in addition to coordinating the process.

### Compliance

Non-adherence to medical therapies is common in chronic disease management and can be further exacerbated during adolescence.^68^ ^69^ Knowledge of medication does not necessarily equate to compliance and forgetting, lack of time, no perceived benefit and too many tablets are common themes in patients with IBD and other conditions. Consultations with the child or young person should focus on devising practical and realistic management plans. ‘Safety netting’ (agreeing a clear plan for what to do if symptoms do not resolve following treatment),^70^ can empower and support the young person in managing their IBD.

## Conclusion

The management of paediatric CD has evolved significantly over recent years with evidence-based guidelines now in place to assist day-to-day practice. Novel tools for personalised IBD management (drug metabolites, anti-TNF antibodies, stool inflammatory markers) provide a better understanding of the patients’ disease status. The pathophysiology of CD remains relatively poorly understood, but advances in molecular technologies are likely to facilitate a deeper understanding of pathways involved in IBD pathogenesis, and will hopefully reveal novel disease biomarkers and treatment strategies.
